# Estimating own-price and cross-price elasticity of cigarette consumption by price tiers in Bangladesh

**DOI:** 10.1136/tc-2022-057679

**Published:** 2023-10-30

**Authors:** Shafiun N Shimul, A K M Ghulam Hussain, Nigar Nargis

**Affiliations:** 1 Institute of Health Economics, University of Dhaka, Dhaka, Bangladesh; 2 Health Policy and Behavioral Sciences, Georgia State University, Atlanta, Georgia, USA; 3 Department of Economics, University of Dhaka, Dhaka, Bangladesh; 4 Tobacco Control Research, American Cancer Society, Atlanta, Georgia, USA

**Keywords:** economics, price, taxation, low/middle income country

## Abstract

**Objectives:**

The overall price elasticity of cigarette consumption in Bangladesh has been studied extensively. The estimates of price elasticity by price tiers are not available in the existing literature.

**Methods:**

Using cohort data of nearly 6000 individuals from the International Tobacco Control Bangladesh survey, this study estimated the own-price and cross-price elasticity and income elasticity of cigarette demand by price tiers in Bangladesh. The elasticity was estimated in three stages of consumer decisions: whether to smoke, which brand to smoke and finally, how many cigarettes to smoke per day. The decision to smoke cigarettes and the choice of cigarette brands were modelled using instrumental variable probability regression. The cigarette consumption per day was modelled using seemingly unrelated regression.

**Results:**

The price elasticity of cigarette smoking prevalence with respect to the price of low-price cigarettes is −0.0487. The total elasticity for low-price cigarette consumption with respect to its own price is −0.1678. The own-price elasticity of smoking intensity of high-priced brands is −0.2512. The cross-price elasticity of low-price cigarette consumption with respect to high-price brand prices is 0.2643. The income elasticity of smoking prevalence overall is 0.0564. The income elasticity of daily consumption of low-price cigarettes is −0.1934 and for high-price cigarettes, it is 1.4044. The total income elasticity is 1.4608 for high-price cigarettes.

**Conclusion:**

A cigarette tax policy that raises the prices of both low-price and high-price brands—but increases prices in the low-price tier at a faster rate than in the high-price tier and increases prices of all brands at a pace faster than income growth—can effectively reduce cigarette consumption in Bangladesh.

**JEL codes:**

H29, L66, I18.

**WHAT IS ALREADY KNOWN ON THIS TOPIC**

- Overall price elasticity of cigarette demand is negative and less than unity.
- Price elasticity of cigarette demand tends to be higher in people with lower socioeconomic status and among youth.

**WHAT THIS STUDY ADDS**

- This is the first study to estimate price elasticity of cigarette demand by price tiers.
- In addition to calculating own-price and income elasticity, this study also quantifies cross-price elasticity by price tiers showing possibility of downward substitution from high-price to low-price brands in the event of price increase.

**HOW THIS STUDY MIGHT AFFECT RESEARCH, PRACTICE OR POLICY**

- The findings from this study hold the potential to facilitate evidence-based tobacco tax modelling, which can be employed to formulate optimal cigarette taxation policies in Bangladesh and simulated for countries with similar complex tiered cigarette tax structure.

## Introduction

Bangladesh has made significant progress in tobacco control since the ratification of the WHO Framework Convention on Tobacco Control in 2005. Several important measures including ban on advertisement and promotion of tobacco products, text and graphic health warnings on packs, tax and price increases, and mass media campaigns to raise public awareness about the harms of tobacco use have contributed to reduction in tobacco use. However, the prevalence of smoking cigarette and bidi among adults in Bangladesh is still very high at around 18%.^1^ Including smokeless tobacco use, overall tobacco use prevalence is 35.3%.^2^ Youth tobacco use is also high—nearly 7% of youths 13–15 years old used tobacco products in 2012.^3^

Even though Bangladesh nearly reaches the minimum international tobacco tax benchmark set by the WHO with a total tax share above 70% of the retail price of the most popular brand of cigarettes, the prices of tobacco products in Bangladesh are among the lowest in the world^3^ and the second lowest (after Myanmar) in the South-East Asia Region.^4^ Hence, high tax share can be misleading as a standalone performance measure of tobacco taxation.^5^

Moreover, Bangladesh experienced relatively high rates of inflation (5.56–12.30%) in the last two decades,^6^ and this inflation was not reflected in higher tobacco product prices. Hence, the real price of tobacco products decreased. High income growth in the recent past, coupled with decreasing real price of tobacco products, increased the affordability of tobacco products, even though in the very recent years, a slight decrease in affordability was also observed.^7^

Furthermore, the effectiveness of any tax increase in Bangladesh is undermined by the presence of a tiered tax system that has at least two inadvertent consequences: (1) the reduction in consumption may be less than intended as tax and price increases may induce smokers to switch to lower-price brands,^5^ and (2) government may not be able to realise the full revenue potential as tobacco companies would likely reposition their brands in lower-tax tiers as a tax avoidance strategy.^8^

It is evident that tobacco industry pricing induces higher cigarette consumption in the low-price tier and undermines the effectiveness of higher taxes in Bangladesh.^9^ Therefore, even though overall tobacco consumption in Bangladesh has been declining,^10 11^ the intensity is increasing (table 1), and a wide price range has kept the option open to brand switching from high-price to low-price cigarettes, especially by those in lower-income groups that may make the target of achieving tobacco-free country by 2040 unattainable.

**Table 1:** Number of adult cigarette smokers, cigarettes per adult and cigarettes per smoker in Bangladesh, 2009 and 2017

	Number of adult cigarette smokers (millions)	Number of cigarettes per adult person per year (sticks)	Number of cigarettes per smoker per year (sticks)
2009	21.90	498	6028
2017	22.35	586	7544

Source: authors’ calculations from total cigarette sales data obtained from the National Board of Revenue, Ministry of Finance, Government of Bangladesh, and number of adult smokers calculated by multiplying total adult population (from the Bangladesh Bureau of Statistics) and adult smoking prevalence (from Global Adult Tobacco Surveys, Bangladesh, 2009 and 2017).

The prevalence of tobacco consumption is much higher among the poorest segment of the population, where the tendency of brand switching to cheaper cigarettes is strong, which is evident from the Global Adult Tobacco Surveys 2009 and 2017 data.^1 10^ Therefore, switching between products or brands of the same product may slow the pace of reducing the overall prevalence of tobacco consumption, especially among the poorest segments of the population. In this connection, it is imperative to understand the brand choice and consumption behaviour of the smoking tobacco users.

In a recent study, Huq *et al* 12 used the International Tobacco Control (ITC) survey data from Bangladesh to model the transition to or from different price tiers of cigarettes. They observed significant movement of smokers across price tiers from one wave to another. The study also investigated the reasons for switching, although no estimation of price elasticity was undertaken for different price tiers. While overall price elasticity estimates are available for cigarette consumption in Bangladesh,^13–15^ the estimates are not yet available by price tiers.

In the absence of estimates of price elasticity by price tiers with differential tax rates, tax simulation analysis, which is necessary to understand the impact of tax policy changes on revenue and consumption, falls short of accuracy in its predictions. This study seeks to fill that knowledge gap by estimating price elasticity by price tiers of cigarette brands.

## Methods

### The data

The data for this study came from four waves of the ITC Bangladesh survey conducted from January 2009 to April 2015 on a cohort of adult tobacco users and non-users. Some variations are observed in the sample size of different waves due to sample attrition and subsequent replenishment (table 2). Altogether, 3245 households responded in all four waves. For 5668 households, data are not available for all waves. The analysis is restricted to male respondents aged 18 years and above, as female smoking prevalence is very low in Bangladesh (less than 2%) and the number of female cigarette smokers in the sample is negligible. The final analytical sample size pooled over the four waves is 8148 observations in an imbalanced panel.

**Table 2:** Sample information of the International Tobacco Control policy evaluation project in Bangladesh: waves 1–4

Wave	Time	Observations	% cigarette smokers
Wave 1	21 January–11 May 2009	5771 (1925 cigarette smokers)	33.36
Wave 2	18 March–5 June 2010	5795 (1971 cigarette smokers)	34.01
Wave 3	16 November 2011–5 June 2012	5522 (1723 cigarette smokers)	31.20
Wave 4	12 October 2014–16 April 2015	4236 (1287 cigarette smokers)	30.38

The dataset includes measures on smoking behaviour of individuals, their sociodemographic characteristics (eg, age, sex, household income, education, occupation and urban/rural area of residence) and purchasing behaviour of tobacco products, including the amount purchased and prices paid. The price and income variables reported in waves 1, 2 and 3 are adjusted for inflation and converted to 2014–2015 (wave 4) constant prices.

### Analytical framework

**Figure 1:**
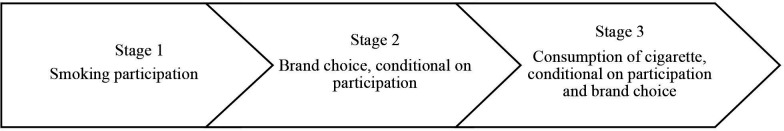
Stages of consumer decision-making in analytical framework.

Based on Marshallian cigarette demand function, we estimated three components of uncompensated own-price and cross-price elasticity pertaining to three stages of consumer decision-making depicted in figure 1: (1) the decision to smoke; (2) choice of low-price or high-price brands, conditional on the decision to smoke; and (3) the number of cigarettes smoked per day, conditional on the decision to smoke and the choice of low-price or high-price brands.

The price elasticity of smoking prevalence was estimated in stage 1 from the information on whether the individual is a cigarette smoker or not. As self-reported price data are only available for those who are smokers, it is necessary to impute the price for non-smokers. In stage 2, the elasticity of choice of low-price versus high-price brands was estimated among the sample of cigarette smokers. In Bangladesh, four types of brands—low, medium, high and premium—are available according to four price tiers. The bottom two tiers (low and medium) were combined into the low-price brand category (henceforth denoted as LM) and the top two tiers (high and premium) were combined into the high-price brand category (henceforth denoted as HP). There are three reasons to make this separation.

First, most of the cigarette consumption in Bangladesh takes place in the low-price and medium-price tiers. Over the period from 2009 to 2015, cigarette sales in the low-price and medium-price tiers accounted for 80–85% of total tax-paid cigarette sales, according to the National Board of Revenue data. Similarly, ITC survey data have far fewer observations on HP brands than on LM brands. Separate analysis for each of the four tiers is not possible due to lack of enough observations in each category. Second, the price variation within each price category is not wide enough to identify the tier-specific effect of price changes on cigarette consumption. Third, tobacco control experts and advocates in Bangladesh have long been recommending for the merger of the bottom two tiers into one low tier and the merger of the top two tiers in one high tier considering the long-term objective of simplification of tax structure. The estimation in this paper follows this convention. There is a reason why experts have made such a recommendation. There is near uniformity of product quality within the low two tiers and the top two tiers which induces brand switching primarily between low and medium tiers and between high and premium tiers. In addition, a study shows that there is very little transition from premium to medium tier in contrast to high transition between low and medium tiers.^12^

In stage 3, the price elasticity of smoking intensity (cigarette consumption per day, or CPD) was estimated separately among the sample of smokers of low-price and high-price brands.

### Empirical models

Three sets of empirical models were applied for each of the three decision stages. In stage 1, to estimate the elasticity of smoking prevalence, a regression of the probability of being cigarette smoker was run on potential determinants of cigarette demand including cigarette prices and consumer income using logit and probit regression models. Since individuals can simultaneously choose consumption level and price, self-reported prices can be endogenous to their consumption decisions which in turn can bias the estimated effect of price. Therefore, prices are instrumented in instrumental variable (IV) probit regression using a composite housing index that summarises characteristics of housing of respondents to represent their socioeconomic status. This composite index was developed in the ITC Bangladesh survey and was categorised into low, medium and high socioeconomic status based on terciles. More details of the housing index are available elsewhere.^16–19^ The housing index is a broad measure of affordability of housing by households and is expected to be highly correlated with the affordability of all household goods and services including tobacco products. Thus, it is expected to reflect the affordability of tobacco products—that is, the price they pay to purchase tobacco products given their household income. The previous study that estimated price elasticity of cigarette consumption using the ITC Bangladesh survey data used tier-specific excise tax rates as the instrument for price.^15^ The same instrument could not be used here since we broke down the analysis by tiers and the tax rates within low or high tier are not variable enough to generate a strong instrument.

In stage 2, to estimate the elasticity of cigarette brand choice, we ran the regression for brand choice (LM vs HP) using logit, probit and IV probit regression models. In stage 3, we applied seemingly unrelated regression (SUR) to estimate the elasticity of smoking intensity. All estimations were appropriately weighted using longitudinal survey weights. The weighted analysis is necessary to correct for the sampling bias resulting from the oversampling of cigarette smokers in the ITC Bangladesh survey and to ensure the national representativeness of the estimates. Using weighted analysis is recommended when sample selection is correlated with the dependent variable (and consequently with the error term).^20^ The sampling weights also adjust the SEs of the estimates for the complex multistage sampling design.

In stage 3, the SUR model is used for CPD or smoking intensity. This model subsumes the second stage as consumption of cigarettes in one of the two price tiers implicitly indicates the choice of brand in that tier. In addition, in the separate regression of brand choice in stage 2, the regression coefficients of the price variables are insignificant. Hence, we report regression results from only stages 1 and 3. The details of the estimation strategy and results are provided in the online supplemental tables A1–A7.

10.1136/tc-2022-057679.supp1 Supplementary data

## Results

Tables 3 and 4 present the summary statistics of the key variables in the analytical sample. On average, cigarette smokers in Bangladesh smoked 10 cigarettes per day, with a high SD. The average price per pack of 20 cigarettes was 60 Bangladeshi taka in 2015 prices. The price gap between the low-price and high-price cigarette brands was significant—the average price of high-price brands was 122 taka, which was nearly three times as high as the average price of low-price brands (table 3). The regressions of self-reported prices of smokers used for imputing prices to non-smokers are shown in the online supplemental table A1. Two-thirds of the sample were located in the rural areas and more than three-quarters of smokers reported smoking low-price brands (table 3).

**Table 3:** Summary statistics of analytical sample

Variable	Observations	Mean	SD	Min	Max
Income (taka in 2015 prices)	8148	41 510	2328	14 750	108 840
Age (years)	8148	40.52	15.72	15.00	108.00
Number of friends who are smokers	8148	3.80	1.46	0.00	5.00
Cigarette consumption per day (sticks)	5847	10.10	10.98	1.00	99.00
Price per pack of 20 cigarette sticks (taka in 2015 prices)	5795	60.44	46.47	10.43	469.03
Price per pack of 20 sticks of low-price cigarettes (taka in 2015 prices)	5795	46.21	14.92	11.76	96.00
Price per pack of high-price cigarettes (taka in 2015 prices)	5795	122.22	32.49	58.56	469.03

**Table 4:** Summary statistics of analytical sample

Variable	Observations	%
Place of residence		
Urban	2177	37.57
Rural	3618	62.43
Price tier		
Low-price brands	4384	75.65
High-price brands	1411	24.34
Marital status		
Unmarried	1015	17.52
Married	4780	82.48
Educational status		
Illiterate	1042	17.98
1–8 years	3150	54.36
9 years or more	1603	27.66
Occupation		
Owner farmer	818	14.12
Tenant farmer	129	2.23
Self-employed in non-farm agricultural activity	1067	18.41
Self-employed in non-agricultural activity	380	6.56
Farm wage labourer	116	2
Non-farm agricultural wage labourer	769	13.27
Non-agricultural wage labourer	89	1.54
Professional (eg, physician, engineer)	286	4.94
Managerial, administrative or clerking	111	1.92
Student	268	4.62
Unemployed	69	1.19
Housewife/housekeeper/household manager	1693	29.21

The IV probit model for the decision to smoke was estimated applying the maximum likelihood estimation method. The model for estimation included the prices of low-price and high-price brands separately (model 2 as shown in the online supplemental table A2). The results of this regression of the decision to smoke presented in table 5 indicate that a 1 taka higher price of low-price cigarettes lowers the probability of smoking by 0.09 percentage points and the number of cigarettes smoked per day by −0.02 for low-price brands. The coefficient of price of HP is statistically insignificant (table 5), that is, the daily cigarette consumption of high-price cigarettes was insensitive to changes in the prices of high-price brands themselves. The validity of the housing index as instrument is tested using the F-statistics of the reduced form regression of cigarette prices of low-price and high-price brands on composite housing index and all other regressors in the IV probit model. The overall F-statistics is greater than 10, indicating that the instruments are strong and identifies the effect of the prices of low-price and high-price brands (note #4 in table 5).

**Table 5:** Results of regression of the decision to smoke and the number of cigarettes smoked per day

	Decision to smoke	Number of cigarettes smoked per day	
	IV probit	Seemingly unrelated regression	
	Smoker	Low-price brands	High-price brands
Price of low-price brands per pack of 20 pieces (taka in 2015 prices)	0.091*** (0.012)	0.022** (0.009)	0.005 (0.006)
Price of high-price brands per pack of 20 pieces (taka in 2015 prices)	0.003 (0.008)	0.018*** (0.004)	0.003 (0.003)
Household income (taka in 2015 prices)	0.018*** (0.004)	0.039*** (0.007)	0.056*** (0.004)
Age (years)	0.005** (0.002)	0.045*** (0.010)	0.028*** (0.006)
Education (reference: illiterate)			
1–8 years	0.202*** (0.055)	0.756** (0.356)	0.435* (0.224)
9 years or more	0.457** (0.193)	3.309*** (0.424)	2.138*** (0.268)
Occupation (reference: owner farmer)			
Tenant farmer	0.173 (0.259)	1.371 (0.922)	0.227 (0.582)
Self-employed in non-farm agricultural activity	0.194** (0.090)	0.529 (0.471)	0.487 (0.297)
Self-employed in non-agricultural activity	0.132 (0.309)	0.507 (0.620)	0.039 (0.391)
Farm wage labourer	0.100 (0.176)	0.585 (0.967)	0.653 (0.610)
Non-farm agricultural wage labourer	0.076 (0.117)	0.138 (0.504)	0.186 (0.318)
Non-agricultural wage labourer	0.829*** (0.132)	2.748** (1.113)	1.568** (0.702)
Professional (eg, physician, engineer)	0.609*** (0.114)	1.608** (0.708)	2.632*** (0.447)
Managerial, administrative or clerking	0.635 (0.469)	0.835 (1.057)	0.371 (0.667)
Student	0.144 (0.118)	0.333 (0.696)	0.094 (0.439)
Unemployed	0.059 (0.308)	2.980** (1.220)	0.083 (0.770)
Housewife/housekeeper/household manager	0.270*** (0.061)	0.220 (0.426)	0.655** (0.269)
Resident of urban area (reference: rural area)	0.311*** (0.075)	2.556*** (0.295)	1.526*** (0.186)
Married	0.159 (0.189)	0.903** (0.379)	0.188 (0.239)
Number of friends who are smokers	0.020 (0.339)	0.654*** (0.107)	0.074 (0.067)
Number of observations	8148	5832	

(1) The z statistics of the coefficients are in parentheses. (2) The levels of significance used are: *p<0.10, **p<0.05, ***p<0.01. (3) The χ2(2) statistics from the Wald test of exogeneity in the IV probit model using prices of low-price and high-price brands instrumented with composite housing index is 6.65 (p=0.0360), which rejects the null hypothesis of exogeneity of cigarette prices at a 5% level of significance. The full set of results including the instrumental variable estimation of cigarette prices is provided in the online supplemental table A2. (4) The validity of the instruments is tested using the F-statistics of the reduced form regression of cigarette prices of low-price and high-price brands on composite housing index and all other regressors in the IV probit model. The overall F-statistics is greater than 10, indicating that the instruments are strong and identifies the effect of the prices of low-price and high-price brands. The results of these regressions are provided in the online supplemental table A3.

IV, instrumental variable.

Both the decision to smoke and the number of cigarettes smoked per day were sensitive to income changes. Higher income led to higher smoking probability overall and greater amount of daily cigarette consumption for high-price brands. However, higher income tends to lower the daily consumption of low-price cigarettes indicating the possibility of upward substitution to higher-price brands as more expensive brands become more affordable with higher income.

Older adults tend to have lower smoking probability overall and lower smoking intensity for high-price brands. The smoking intensity of low-price brands tends to get higher at older age. Overall smoking probability is higher among more educated persons. Smoking intensity tends to be lower among higher-educated smokers for low-price brands and higher for high-price brands. Residents of urban areas tend to have higher smoking probability and intensity of smoking high-price brands and lower intensity of smoking low-price brands. Smokers who are married and have more friends who are smokers demonstrate higher intensity of smoking low-price cigarettes. Some profession-specific differences are also observed in smoking probability and intensity.

Because we observe endogeneity of self-reported prices as the null of exogeneity is rejected (Χ^2^=6.65, p=0.036), only the price coefficients estimated from the IV probit regression are reliable and hence these results have been used in the estimation of own-price and cross-price elasticity of cigarette smoking prevalence presented in table 6.

**Table 6:** Own-price and cross-price elasticity and income elasticity estimates of cigarette demand by low-price and high-price tiers

	Smoking prevalence	Smoking intensity		Total elasticity (low-price brands)	Total elasticity (high-price brands)
		Low-price brands	High-price brands		
Price of low-price brands	0.0487** (0.0150)	0.1191** (0.0489)	0.1335 (0.1551)	0.1678	–
Price of high-price brands	0.0318 (0.1567)	0.2643* (0.0619)	0.2512 (0.1994)	0.2643	0.2512
Income	0.0564* (0.0780)	0.1934** (0.0334)	1.4044* (0.1066)	0.1370	1.4608

(1) The SEs are in parentheses. The level of significance using two-tailed test is: *p<0.10, **p<0.05, ***p<0.01. (2) Total elasticity is estimated by summing only significant coefficients across smoking prevalence and smoking intensity. (3) For smoking prevalence, IV probit regression model coefficients are used. (4) For smoking intensity, SUR regression coefficients are used. (5) The coefficients that were not statistically significant are not used for the calculation of total elasticity.

*Indicates that even though income coefficient was significant in the IV probit regression in table 5, the marginal effect at the mean income is found to be insignificant. Nevertheless, it is considered in overall elasticity calculation.

IV, instrumental variable; SUR, seemingly unrelated regression.

The independent regressions of the choice of low-price versus high-price brands using logit, panel logit, probit, panel probit and IV probit estimations do not provide any statistically significant estimates of the effects of price on brand choice (online supplemental table A6). However, higher income tends to lower the probability of choosing low-price brands. Similarly, the independent regressions for CPD for low-price and high-price brands using pooled ordinary least squares regression, panel regression or two-stage least squares regression do not provide any statistically significant estimate of the effects of cigarette price on daily cigarette consumption (online supplemental table A7). However, the SUR model does provide meaningful estimates of the relationship of own-price and cross-price elasticity of low-price and high-price brands with the intensity of smoking each type of brand, as indicated in the discussion of the results in table 5.

Table 6 shows estimates of price and income elasticity based on the coefficients of price and income variables in the IV probit and SUR regressions for the decision to smoke and cigarettes smoked per day. The elasticity is calculated at the sample mean values of price and income. The total elasticity is given by the sum of the elasticity of smoking prevalence and the elasticity of smoking intensity.

The price elasticity of smoking prevalence with respect to the price of low-price brands is estimated at −0.0487 (p<0.01). The total elasticity for low-price cigarette consumption with respect to its own price is −0.1678, which is the sum of the elasticity of smoking prevalence of −0.0487 (p<0.01) and the elasticity of smoking intensity of −0.1191 (p<0.01). It implies that a 10% increase in the price of low-price cigarettes is expected to lead to 0.487% reduction in cigarette smoking prevalence and a 1.191% decrease in daily consumption of low-price cigarettes, with a total of 1.678% reduction in the consumption of low-price cigarettes. As higher prices of low-price brands lead to lower smoking prevalence overall, it is expected to reduce the likelihood of smoking across all brands including low-price and high-price brands.

Smoking prevalence is not sensitive to increases in the price of high-price brand cigarettes. Smoking intensity of high-price cigarette smokers is not sensitive to the changes in its own price upon consideration of the statistical significance of the price elasticity estimate of −0.2512 (p<0.10) at a 10% level using a two-tailed test. With a one-tailed test, this estimate can, however, be considered significant at the 10% level. This estimate indicates that a 10% increase in the price of high-price brands leads to a reduction in the smoking intensity of high-price brand smokers by 2.512%. Increases in the price of high-price cigarettes by 10% may induce smokers to switch to low-price cigarettes increasing low-price cigarette consumption by 2.643%, as indicated by the cross-price elasticity of low-price cigarette consumption with respect to high-price brand prices at −0.2643 (p<0.0619).

The income elasticity of smoking prevalence overall is 0.0564 (p<0.07), suggesting that a 10% increase in income may lead to a 0.564% increase in cigarette smoking prevalence. The income elasticity of daily consumption of low-price cigarettes is −0.1934 (p=0.0334) and for high-price cigarettes, it is 1.4044, which indicate that income growth can lead to reduction in the intensity of smoking low-price cigarettes and increase in the intensity of smoking high-price cigarettes. The total income elasticity is negative for low-price cigarettes as the negative effect of income growth on smoking intensity is greater than the positive effect on smoking prevalence. The total income elasticity is 1.4608 for high-price cigarettes, signifying that higher income enables smokers to purchase more expensive brands and therefore increases the demand for high-price cigarettes.

## Discussion

Differentiating price sensitivity of smoking decisions by brands or price tiers is nearly absent in the existing literature, apart from a study for China.^21^ This study makes an important contribution to the literature by examining the price sensitivity of the decision to smoke cigarettes, the selection of low-price or high-price brands, and the number of cigarettes smoked per day by price tiers of cigarettes. The price elasticity of smoking prevalence in Bangladesh with respect to the price of low-price brands was estimated at −0.0487. Although this estimate is relatively low compared with the estimates available from previous studies in Bangladesh, it is not comparable with earlier estimates due to differences in estimation methods. Most of the studies estimating price sensitivity of cigarette consumption focus on overall price elasticity of cigarette demand. The price elasticity estimate based on the prices of low-price brands is expected to be lower than the estimate based on all brand prices because of the large price difference between low-price and high-price brands (46.21 taka vs 122.22 taka, as shown in table 3).

The elasticity of smoking intensity of low-price and high-price cigarettes with respect to own prices was −0.1191 and −0.2512, respectively, suggesting that both low-price and high-price brand smokers respond to price increases by reducing daily consumption. Increasing price in the low-price tier is crucial for reducing smoking prevalence, as smoking prevalence is sensitive to low-price brand price changes only, which is expected given the high volume and market share (80–85%) of low-price cigarettes in Bangladesh.

The positive cross-price elasticity of daily cigarette consumption of low-price cigarettes with respect to high-price brands at 0.2643 provides evidence of downward substitution from high-price tiers to low-price tiers. The existence of a high price differential and the tiered tax structure in Bangladesh is favourable to downward substitution and can undermine the effectiveness of tax and price increases in reducing overall cigarette consumption. The findings of this study are relevant and timely for Bangladesh, which has been burdened with a tiered tax and price structure for cigarettes for decades, thereby inhibiting the tremendous potential of cigarette taxation, which is proven to be one of the most effective tobacco control measures worldwide.

The income elasticity of smoking prevalence overall is 0.0564, indicating that income growth can induce more people to smoke. However, the negative income elasticity of daily consumption of low-price cigarettes and positive income elasticity of daily cigarette consumption of high-price cigarettes indicate that higher income may lead smokers to substitute upward and purchase more expensive brands. This finding is consistent with a previous study that observed upward substitution by Bangladeshi smokers.^12^

The current study findings have strong policy implications. First, a price increase only in the high-price tier—if not increased in all brand types— will not reduce the prevalence of smoking or daily cigarette consumption of high-price cigarettes. Instead, it will induce smokers to switch to lower-price brands. For instance, in Bangladesh, from 2019–2020 to 2020–2021, total cigarette sales increased by 5.2%, largely driven by an 11.1% increase in the sales of low-price cigarette brands. This increase was partially offset by decreases in sales in high-price (−0.2%) and medium-price tiers (−25.8%), while sales in the premium tier continued to increase (5.6%). These patterns of change in sales in different price tiers are consistent with a positive and significant cross-price elasticity of low-price brand cigarettes and price insensitivity of high-price brands.

Second, the price gap between low-price and high-price tiers should be narrowed down over time to minimise the incentive to substitute to cheaper brands when prices increase. This would require increases in cigarette prices in the low-price tier that are faster than increases in the high-price tier for gradual convergence of prices. Introduction of specific taxes and simplification of the current four-tiered tax structure into a uniform specific system would help reduce the price gap further.

Third, income growth can induce higher smoking prevalence unless cigarette prices increase significantly to outpace income growth and reduce the affordability of cigarettes. The affordability of cigarettes increased in Bangladesh over 2009–2015, due to fast income growth and modest increases in cigarette prices.^5^ While formulating cigarette tax policy reforms, it is important to increase taxes and prices enough to exceed income growth after adjustment for inflation.

Fourth, the revenue authority of the government typically hesitates to increase tax on cigarettes fearing that it would have an adverse effect on their revenue collection. However, the price elasticity of cigarette consumption in the low-price tier is less than one and very small, meaning that the percentage decrease in cigarette sales will be far less than the percentage increase in price and tax, and total revenue is expected to increase significantly following a tax-induced price increase.

One major limitation of the study is that the analytical sample used for the estimation of price sensitivity of cigarette consumption is limited to adult men aged 18 years and above and may not necessarily represent the price responsiveness of youth and women. Since smoking prevalence is insignificant among women (less than 2%) in Bangladesh, the estimates from this study do not lose national representation of the adult population by excluding female respondents. Even though tobacco market dynamics involve current and previous bidi smokers, the current study is limited to only cigarette smokers for estimating cigarette demand elasticity with respect to changes in cigarette prices. A separate study is needed to examine the movement of bidi smokers in and out of the cigarette market specifically in the low tier.

In recent years, there has been dramatic movement of users between medium and lower price tier cigarettes in Bangladesh. However, our current study could not capture those dynamics as data do not permit analysis for recent years especially since 2017. Moreover, tobacco industries often reposition their market strategies by shifting focus from one tier of product to other tiers, and hence, supply-side interventions also play a role. However, our current study focuses more on the demand side and does not factor industry strategies into the analysis. This topic has been dealt with in a previous paper based on the same dataset.^9^

This study assumes uniform price elasticity of cigarette demand across population subgroups and over time. Price elasticity can be sensitive to various factors, such as availability of substitutes, type of the commodity in question, budget share spent on the commodity, consumer preference or the time elapsed since the price changed. We could not test the sensitivity of price elasticity to any of these factors due to the small sample size particularly at the high-price tier when the data are divided by waves and lack of sufficient price variability within low or higher-price tier in each wave. For example, smoking prevalence among women is very low (<2%) and the sample size of female smokers is too small (n=193; only 2.13% of the samples of cigarette smokers) to conduct the analysis by sex. Previous studies disaggregated the analysis by socioeconomic status based on the housing index. Since the analysis in this paper is already disaggregated by price tiers, it is unrealistic to allow further disaggregation of analysis in consideration of the loss of statistical power from small sample size. Nevertheless, there might be heterogeneity of price sensitivity by price tiers across different population subgroups which remains to be addressed in future research based on a larger sample size.

Finally, this study does not include the potential behavioural response due to policy changes other than prices, such as smoke-free laws, ban on advertising, promotion and sponsorship, cessation support programmes, warning labels and the like. Incorporating all the policy variables in the analysis would provide a more comprehensive understanding of how the response to price changes interacts with other interventions. However, the tobacco control policy environment in Bangladesh did not experience any remarkable shift in the non-tax policy interventions over the period under study. Hence, we are unable to control for the comprehensive tobacco control policy measures in this paper although the ITC survey did ask questions to measure the salience of other policy measures. It can be the subject of future investigation with a longer observation period that includes significant policy changes.

### Conclusion

Increasing the price of low-price cigarette brands can effectively reduce smoking prevalence and the daily cigarette consumption of smokers, thereby reducing overall cigarette consumption. Increasing the price of high-price cigarette brands without increasing the prices of low-price brands may encourage smokers to switch to low-price brands instead of quitting. As income growth contributes to higher smoking prevalence, increases in cigarette prices need to outpace income growth and inflation. A cigarette tax policy that raises the prices of both low-price and high-price brands increasing prices in the low-price tier at a faster rate than in the high-price tier and increasing prices of all brands at a pace faster than income growth can effectively reduce cigarette consumption in Bangladesh.

## Data Availability

Data may be obtained from a third party and are not publicly available. The dataset is collected from the International Tobacco Control Policy Evaluation (ITC) project and data is not publicly available. Researchers can request data from ITC project using the form available on the website (https://itcproject.org/request-data-form/)
