# Supplementary material for: Estimating own-price and cross-price elasticity of cigarette consumption by price tiers in Bangladesh
Source: Tob Control. 2023 Oct 30;33(Suppl 2):s44–50. doi: 10.1136/tc-2022-057679 (PMC11058103; doi:10.1136/tc-2022-057679)
Supplement: Supplementary data [file tc-2022-057679supp001.pdf]

Supplement

A. Empirical strategy

Stage 1: Smoking prevalence

To estimate the elasticity of smoking prevalence, a regression of the probability of being a cigarette smoker is first run on the potential determinants of cigarette demand including price, income, individual socio-demographics, and other factors. The models are specified as follows:

$$\Pr(Smoker_{it} = 1 | P_{it}, Z_{it}, \in_{it}) = \alpha_i + \beta_o P_{it} + \beta_i Z_{it} + \in_{it} \quad \text{(Model 1)}$$

$$\Pr\left(Smoker_{it} = 1 \middle| P_{it}^{LM}, P_{it}^{HP}, Z_{it}, \in_{it}'\right) = \alpha'_i + \beta_{LM} P_{it}^{LM} + \beta_{HP} P_{it}^{HP} + \beta'_i Z_{it} + \in'_{it} \quad \text{(Model 2)}$$

where *P* is price and *Z* is a vector of the other control variables of income, age, education level, occupation, rural/urban resident status, marital status, and the number of friends who are smokers. The suffix *i* stands for individual respondent and *t* stands for waves 1, 2, 3, and 4. While model (1) controls for a single price, model (2) includes prices of LM and HP brands (P_{it}^{LM}, P_{it}^{HP}) separately. Model (1) shows whether overall price matters for the decision to smoke. Model (2) captures the effect of prices on smoking prevalence at different price segments. In both models, the other control variables remain the same.

Since individuals can simultaneously choose consumption level and price, self-reported prices can be endogenous to their consumption decisions which in turn can bias the estimated effect of price. Therefore, prices are instrumented using a composite housing index that summarizes characteristics of housing of respondents to represent their socioeconomic status. This composite index was developed in the ITC Bangladesh survey and was categorized into low, medium, and high socioeconomic status based on terciles. More details of the housing index are available in the *ITC Project Technical Reports* (ITC Project Wave 1-Wave 4). The housing index is a broad measure of affordability of housing by households and is expected to be highly correlated with the affordability of all household goods and services including tobacco products. Thus, it is expected to reflect the affordability of tobacco products—that is, the price they pay to purchase tobacco products given their household income. Considering that the “number of friends” variable may be endogenous, the regression is also run without the variable. No changes are observed in the statistical significance of other variables. Although the magnitude of the estimates changes to some extent, they are not statistically different from the estimates obtained from the regression that includes the “number of friends who are a smoker” variable.

The abovementioned models are estimated using logit, probit, panel logit, panel probit, and instrumental variable probit (IV probit) regressions. In panel logit and probit estimation, random effects models are used instead of fixed effects models because: (i) the fixed effects models would lose observations for those respondents who appear only once in the panel and (ii) the fixed effects model would not allow for the identification of the effect of any variable with no within-person variation (e.g., residence) or little within-person variation (e.g., completed education) over time.

As the purchase price of cigarettes was not reported by non-smokers, their price is imputed using predicted price from the following random effects regression models of self-reported price for all brands and for LM and HP separately:

$$\mathrm{P}_{it} = \gamma_o + \gamma_1 Z''_{it} + v_i + wave_t + psu_{is} + \varepsilon''_{it} \qquad (\mathrm{I}_o)$$

$$P_{it}^{LM} = \gamma_0^{LM} + \gamma_1^{LM} Z''_{it} + v_i + wave_t + psu_{is} + \varepsilon''_{it} \qquad (\mathrm{I}_1)$$

$$P_{it}^{HP} = \gamma_0^{HP} + \gamma_1^{HP} Z''_{it} + v_i + wave_t + psu_{is} + \varepsilon''_{it} \qquad (\mathrm{I}_2)$$

where P_{it} is the self-reported price of smokers, which is categorized into P_{it}^{LM} and P_{it}^{HP} based on the brand names reported by smokers and corresponding price tiers specified in the cigarette tax data provided by the National Board of Revenue. v_i is the random effect corresponding to individual *i* and ε''_{it} is the random error term. The variable wave stands for the time effect in each wave. The dummy variables *psu* stand for the primary sampling unit *s* representing the location of respondent *i*. Z''_{it} is the vector of exogeneous variables that include income, age, education level, occupation, rural/urban resident status, marital status, and the number of friends who are smokers.

As the price regression is used for out-of-sample prediction of price for non-smokers that cannot be carried out with fixed effects regression, equations (I_o), (I_1), and (I_2) are estimated using random effects estimation.

The predicted prices $\widehat{P_{it}}, \widehat{P_{it}^{LM}}, \widehat{P_{it}^{HP}},$ from equations (I_o), (I_1), and (I_2), respectively, are then imputed to non-smokers in the regression of smoking prevalence in models (1) and (2).

Stage 2: Brand choice (BC)

The brand choice regression model used in this study is specified as follows:

$$P(BC_{it}^{LM} = 1 | P_{it}^{LM}, P_{it}^{HP}, Z_{it}, \in_{it}^{LM}) = a_i^{LM} + \beta_{LM} P_{it}^{LM} + \beta_{HP} P_{it}^{HP} + \beta'_i Z_{it} + \in_{it}^{LM} \quad \text{(Model 3)}$$

where BC_{it}^{LM} = 1 if an individual smoker reported smoking a low-price brand (LM) and 0 if an individual smoker reported smoking a high-price brand (HP). The control variables (Z_{it}) include income, age, education level, occupation, rural/urban resident status, marital status, and the number of friends who are smokers. Model (3) is estimated using logit, probit, panel logit, panel probit, and finally IV probit (based on composite housing index as an instrument) estimation.

Stage 3: Number of cigarettes smoked per day (CPD)

The smoking intensity of smokers is modeled using the following four regression equations:

$$\mathrm{CPD}_{it} = \alpha''_i + \beta''_o P_{it} + \beta''_i Z_{it} + \in''_{it} \quad \text{(Model 4)}$$

$$\mathrm{CPD}_{it} = \alpha''_i + \beta''' P_{it}^{LM} + \beta''' P_{it}^{HP} + \beta''_i Z_{it} + \in''_{it} \quad \text{(Model 5)}$$

$$CPD_{it}^{LM} = \alpha_i^{LM} + \beta^{LM} P_{it}^{LM} + \beta^{HP} P_{it}^{HP} + \beta'_{LM} Z_{it} + \in^{LM}{}_{it} \quad \text{(Model 6)}$$

$$CPD_{it}^{HP} = \alpha_i^{HP} + \beta'^{LM} P_{it}^{LM} + \beta'^{HP} P_{it}^{HP} + \beta''_{HP} Z_{it} + \in^{HP}{}_{it} \quad \text{(Model 7)}$$

The right-hand-side variables in each model are the same as in stage 2. Models (4) and (5) do not distinguish between the CPD of LM and HP brands for all smokers. In model (4) a single price variable is included, and in model (5) the prices of both LM and HP brands are included. Models (6) and (7) are used to run separate regressions for LM and HP brand categories. Both models (6) and (7) use the prices of LM and HP separately. To circumvent potential endogeneity of self-reported prices, along with pooled ordinary least squares regression and panel regressions, two-stage least squared regression models are used.

CPD_{it}^{LM} and CPD_{it}^{HP} are likely to be correlated, as the consumption of one type will reduce the likelihood of using the other type, and hence the error terms of these regressions ($\in^{LM}{}_{it}$ and $\in^{HP}{}_{it}$) are likely to be correlated. Therefore, a seemingly unrelated regression is also estimated that incorporates both the choice of brands (LM versus HP) and CPD corresponding to each brand type. This step combines the decisions of brand choice and CPD in one regression and compares the results with the regressions run independently in stages 2 and 3 above. A similar approach was taken in Stoklosa et al. (2017), based on the ITC survey data for Zambia, to estimate the price elasticity of smoking prevalence. That study found the price elasticity of smoking prevalence for machine-made cigarettes was -0.20, and for roll-your-own cigarettes it was -o.03 when they were estimated taking the simultaneity of smoking both types of cigarettes into account.

To make the estimates nationally representative, all regressions are weighted based on cross-sectional sampling weights that allow for complex multi-stage probability sampling design. Since smoking prevalence cannot be separated for low-price and high-price cigarette smoking, own-price elasticity of

smoking prevalence is only estimated with respect to the prices of low-price and high-price brands and income elasticity of smoking prevalence. It is not possible to estimate cross-price elasticity of smoking prevalence. From the models of smoking intensity, both own- and cross-price elasticity and income elasticity of low-price and high-price cigarette brands are estimated. The elasticities are calculated at the mean prices and income, based on the most reliable set of estimates of marginal effects of price and income on cigarette demand.

B. Tables

Table A1. Results of random effects regressions of cigarette price for all, low-price brands, and high-price brands

	Price	Price of low-price brands	Price of high-price brands
**Income (in thousand taka in 2015 prices)**	0.404***	0.129***	0.170***
	(0.025)	(0.014)	(0.051)
**Age (years)**	-0.286***	-0.094***	-0.048
	(0.040)	(0.018)	(0.110)
**Education (Reference: illiterate)**			
**1 to 8 years**	4.816***	2.835***	4.035
	(1.423)	(0.635)	(5.641)
**9 years or more**	21.566***	5.175***	8.152
	(1.739)	(0.838)	(5.710)
**Occupation (Reference: owner farmer)**			
**Tenant farmer**	4.695	1.080	-3.045
	(3.247)	(1.670)	(8.693)
**Self-employed in non-farm agricultural**	4.610***	2.523***	-0.160
	(1.731)	(0.841)	(6.131)
**Self-employed in non-agricultural activity**	0.536	-1.305	27.312**
	(2.121)	(0.985)	(12.829)
**Farm wage laborer**	-5.139	2.153	-1.272
	(3.377)	(1.582)	(15.034)
**Non-farm agricultural wage laborer**	-2.674	1.112	-6.160
	(1.861)	(0.892)	(6.621)
**Non-agricultural wage laborer**	23.976***	7.677***	6.759
	(4.067)	(2.969)	(7.779)
**Professional (e.g., physician, engineer)**	17.706***	4.886***	4.823
	(2.570)	(1.627)	(6.557)
**Managerial, administrative, or clerking**	7.909**	6.972***	-5.559
	(3.796)	(2.696)	(7.720)
**Student**	1.145	2.623**	-7.578
	(2.464)	(1.234)	(7.360)
**Unemployed**	-4.206	-0.916	-3.252
	(4.104)	(2.020)	(13.642)
**Housewife/Housekeeper/Household manager**	6.526***	3.898***	-0.665
	(1.593)	(0.752)	(6.015)
**Resident of urban area (Reference: rural area)**	8.089***	-0.234	-2.675
	(1.897)	(0.830)	(6.102)
**Married**	-4.742***	-2.156***	0.074
	(1.401)	(0.731)	(2.926)
**Number of friends who are smokers**	-0.122	0.149	0.345
	(0.372)	(0.196)	(0.856)
**Wave (Reference: wave 1)**			
**Wave 2**	0.863	3.002***	-3.263
	(1.155)	(0.623)	(2.844)
**Wave 3**	13.387***	8.836***	39.453***
	(1.234)	(0.649)	(3.150)
**Wave 4**	15.343***	9.138***	45.368***
	(1.289)	(0.677)	(3.130)
**Observations**	6,115	4,552	1,474

Notes:
1. Standard errors in parentheses.
2. * $p < .10$, ** $p < .05$, *** $p < .01$.

3. The coefficients for primary sampling unit location variables are omitted for brevity of presentation.

Table A2. Results of IV probit estimation for the decision to smoke

	IV probit: Decision to smoke	First stage: Low-price brands	First stage: High-price brands
Price of low-price brands per pack of 20 cigarettes (taka in 2015 prices)	-0.091***		
	(0.012)		
Price of high-price brands per pack of 20 cigarettes (taka in 2015 prices)	-0.003		
	(0.008)		
Household income (taka in 2015 prices)	0.018***	0.181***	0.581***
	(0.004)	(0.008)	(0.026)
Age (years)	-0.005**	-0.053***	0.069
	(0.002)	(0.013)	(0.049)
Education (Reference: illiterate)			
1 to 8 years	0.202***	2.165***	-0.086
	(0.055)	(0.518)	(1.839)
9 years or more	0.457**	4.978***	5.290**
	(0.193)	(0.592)	(2.160)
Occupation (Reference: owner farmer)			
Tenant farmer	0.173	1.853	9.361***
	(0.259)	(1.242)	(3.006)
Self-employed in non-farm agricultural activity	0.194**	1.928***	4.262*
	(0.090)	(0.678)	(2.429)
Self-employed in non-agricultural activity	-0.132	-2.375***	28.380***
	(0.309)	(0.768)	(2.060)
Farm wage laborer	0.100	1.009	6.645*
	(0.176)	(1.405)	(3.616)
Non-farm agricultural wage laborer	0.076	0.694	1.047
	(0.117)	(0.731)	(1.917)
Non-agricultural wage laborer	0.829***	8.906***	4.779
	(0.132)	(1.139)	(3.951)
Professional (e.g., physician, engineer)	0.609***	6.213***	12.155***
	(0.114)	(0.902)	(2.496)
Managerial, administrative, or clerking	0.635	7.447***	-5.428
	(0.469)	(0.982)	(4.236)
Student	0.144	1.844*	-10.487***
	(0.118)	(0.945)	(2.590)
Unemployed	-0.059	-0.282	-2.567
	(0.308)	(0.929)	(3.030)
Housewife/Housekeeper/ Household manager	0.270***	2.961***	-1.389
	(0.061)	(0.586)	(1.912)
Resident of urban area (Reference: rural area)	0.311***	3.399***	-3.261**
	(0.075)	(0.415)	(1.473)

Married	-0.159	-1.927***	1.486
	(0.189)	(0.501)	(1.475)
Number of friends who are	-0.020	-0.461***	-2.863***
smokers	(0.339)	(0.108)	(0.356)
Composite housing index			
(Reference: low)			
Moderate		0.012	-1.516
		(0.319)	(1.433)
High		0.142	-5.689***
		(0.427)	(1.476)
Observations	8148	8148	8148
Wald test of exogeneity:			
chi2(2)	6.65		
Prob > Chi^2	0.0360		

Notes:
1. Standard errors in parentheses.
2. * p < .10, ** p < .05, *** p < .01.

Table A3. Results of reduced-form regressions of the prices of low-price and high-price cigarette brands

	First stage of low-price brands	First stage of high-price brands
Household income (taka in 2015 prices)	0.181***	0.581***
	(0.008)	(0.026)
Age (years)	-0.053***	0.068
	(0.013)	(0.049)
Education (Reference: illiterate)		
1 to 8 years	2.164***	-0.089
	(0.519)	(1.840)
9 years or more	4.979***	5.325**
	(0.592)	(2.161)
Occupation (Reference: owner farmer)		
Tenant farmer	1.853	9.370***
	(1.243)	(3.009)
Self-employed in non-farm agricultural activity	1.928***	4.246*
	(0.679)	(2.428)
Self-employed in non-agricultural activity	-2.375***	28.398***
	(0.770)	(2.059)
Farm wage laborer	1.010	6.660*
	(1.407)	(3.623)
Non-farm agricultural wage laborer	0.695	0.981
	(0.734)	(1.917)
Non-agricultural wage laborer	8.905***	4.751
	(1.140)	(3.955)
Professional (e.g., physician, engineer)	6.214***	11.934***
	(0.903)	(2.502)
Managerial, administrative, or clerking	7.448***	-5.456
	(0.983)	(4.241)
Student	1.845*	-10.473***
	(0.946)	(2.591)
Unemployed	-0.282	-2.588
	(0.930)	(3.033)
Housewife/Housekeeper/ Household manager	2.960***	-1.395
	(0.587)	(1.912)
Resident of urban area (Reference: rural area)	3.398***	-3.255**
	(0.416)	(1.474)
Married	-1.925***	1.514
	(0.501)	(1.475)
Number of friends who are smokers	-0.461***	-2.878***
	(0.108)	(0.357)
Composite housing index (Reference: low)		
Moderate	0.010	-1.475
	(0.417)	(1.470)
High	0.142	-5.677***
	(0.448)	(1.485)
Observations	8,151	8,166
F-statistic (overall with p-value)	105 (0.000)	65 (0.000)
F-statistic (for instruments with p-value in parentheses)	0.06 (0.93)	8.06 (0.003)

Notes:
1. Standard errors in parentheses.

2. * p < .10, ** p < .05, *** p < .01.

Table A4. Results of regressions of the decision to smoke based on the cigarette price variable for all brands

	Logit	Panel-logit	Probit	Panel-probit	IV probit
Price per pack of 20 cigarettes (taka in 2015 prices)	-0.001	0.000	-0.001	0.000	-0.003
	(0.002)	(0.002)	(0.001)	(0.002)	(0.037)
Income (taka in 2015 prices)	0.006*	-0.001	0.003*	-0.001	0.004
	(0.003)	(0.003)	(0.002)	(0.003)	(0.023)
Age (years)	-0.005	-0.021***	-0.003	-0.021***	-0.003
	(0.005)	(0.005)	(0.003)	(0.005)	(0.006)
Education (Reference: illiterate)					0.000
					(.)
1 to 8 years	0.107	0.347**	0.068	0.347**	0.075
	(0.187)	(0.164)	(0.110)	(0.164)	(0.152)
9 years or more	-0.282	0.190	-0.167	0.190	-0.131
	(0.231)	(0.200)	(0.135)	(0.200)	(0.777)
Occupation (Reference: owner farmer)					0.000
					(.)
Tenant farmer	-0.573	0.508	-0.332	0.508	-0.319
	(0.412)	(0.350)	(0.242)	(0.350)	(0.340)
Self-employed in non-farm	0.138	0.548***	0.083	0.548***	0.096
agricultural	(0.230)	(0.189)	(0.133)	(0.189)	(0.276)
Self-employed in non-agricultural	0.100	0.009	0.063	0.009	0.063
activity	(0.216)	(0.221)	(0.128)	(0.221)	(0.122)
Farm wage laborer	-0.298	0.787**	-0.180	0.787**	-0.186
	(0.350)	(0.393)	(0.213)	(0.393)	(0.255)
Non-farm agricultural wage laborer	0.215	0.851***	0.127	0.851***	0.129
	(0.207)	(0.207)	(0.123)	(0.207)	(0.113)
Non-agricultural wage laborer	0.076	-0.353	0.043	-0.353	0.092
	(0.381)	(0.429)	(0.226)	(0.429)	(1.025)
Professional (e.g., physician,	0.184	0.484*	0.113	0.484*	0.152
engineer)	(0.284)	(0.289)	(0.165)	(0.289)	(0.818)
Managerial, administrative, or	-0.752**	-1.023***	-0.449**	-1.023***	-0.432
clerking	(0.326)	(0.379)	(0.185)	(0.379)	(0.384)
Student	0.090	0.101	0.065	0.101	0.070
	(0.282)	(0.254)	(0.162)	(0.254)	(0.180)
Unemployed	-0.628**	-1.045***	-0.382**	-1.045***	-0.386**
	(0.282)	(0.342)	(0.157)	(0.342)	(0.177)
Housewife/Housekeeper/	0.072	0.253	0.049	0.253	0.063
Household manager	(0.185)	(0.168)	(0.110)	(0.168)	(0.309)
Resident of urban (Reference: rural area)	0.240	0.639***	0.141	0.639***	0.164
	(0.153)	(0.167)	(0.091)	(0.167)	(0.519)
Married	0.325**	0.608***	0.191**	0.608***	0.183
	(0.164)	(0.159)	(0.096)	(0.159)	(0.195)
Number of friends who are smokers	0.756***	0.863***	0.448***	0.863***	0.445***
	(0.040)	(0.038)	(0.022)	(0.038)	(0.074)
Observations	8,148	8,150	8,148	8,150	8,148
Pseudo R^2	0.213		0.213		

Notes:
1. The z statistics of the coefficients are in parentheses.
2. * p < .10, ** p < .05, *** p < .01
3. The Wald statistics from IV probit regression using composite housing index as an instrument for the cigarette price for all brands are not statistically significant. Hence, the exogeneity of price is not rejected.

Table A5. Results of regressions of the decision to smoke based on the cigarette price variable for low-price and high-price brands

	Logit	Panel-logit	Probit	Panel-probit	IV probit
Price of low-price brands per pack of 20 cigarettes (taka in 2015 prices)	0.005	-0.001	0.003	0.005	-0.091***
	(0.004)	(0.004)	(0.002)	(0.004)	(0.012)
Price of high-price brands per pack of 20 cigarettes (taka in 2015 prices)	-0.001	-0.002	-0.000	-0.001	-0.003
	(0.002)	(0.002)	(0.001)	(0.002)	(0.008)
Household income (taka in 2015 prices)	0.004	0.000	0.003	0.004	0.018***
	(0.003)	(0.003)	(0.002)	(0.003)	(0.004)
Age (years)	-0.004	-0.021***	-0.002	-0.004	-0.005**
	(0.005)	(0.005)	(0.003)	(0.005)	(0.002)
Education (Reference: illiterate)					
1 to 8 years	0.089	0.356**	0.058	0.089	0.202***
	(0.187)	(0.164)	(0.110)	(0.187)	(0.055)
9 years or more	-0.338	0.209	-0.201	-0.338	0.457**
	(0.228)	(0.198)	(0.133)	(0.228)	(0.193)
Occupation (Reference: owner farmer)					
Tenant farmer	-0.583	0.535	-0.339	-0.583	0.173
	(0.418)	(0.352)	(0.245)	(0.418)	(0.259)
Self-employed in non-farm agricultural activity	0.124	0.568***	0.073	0.124	0.194**
	(0.232)	(0.190)	(0.134)	(0.232)	(0.090)
Self-employed in non-agricultural activity	0.144	0.062	0.085	0.144	-0.132
	(0.223)	(0.229)	(0.131)	(0.223)	(0.309)
Farm wage laborer	-0.287	0.805**	-0.174	-0.287	0.100
	(0.351)	(0.394)	(0.213)	(0.351)	(0.176)
Non-farm agricultural wage laborer	0.214	0.862***	0.125	0.214	0.076
	(0.208)	(0.208)	(0.124)	(0.208)	(0.117)
Non-agricultural wage laborer	-0.002	-0.312	-0.006	-0.002	0.829***
	(0.379)	(0.429)	(0.226)	(0.379)	(0.132)
Professional (e.g., physician, engineer)	0.133	0.521*	0.080	0.133	0.609***
	(0.285)	(0.289)	(0.165)	(0.285)	(0.114)
Managerial, administrative, or clerking	-0.805**	-1.013***	-0.481***	-0.805**	0.635
	(0.325)	(0.380)	(0.184)	(0.325)	(0.469)
Student	0.070	0.092	0.053	0.070	0.144
	(0.285)	(0.255)	(0.164)	(0.285)	(0.118)
Unemployed	-0.622**	-1.034***	-0.378**	-0.622**	-0.059
	(0.284)	(0.343)	(0.158)	(0.284)	(0.308)
Housewife/Housekeeper/ Household manager	0.047	0.259	0.033	0.047	0.270***
	(0.188)	(0.169)	(0.112)	(0.188)	(0.061)
Resident of urban area (Reference: rural area)	0.199	0.641***	0.116	0.199	0.311***
	(0.154)	(0.165)	(0.092)	(0.154)	(0.075)
Married	0.344**	0.615***	0.202**	0.344**	-0.159
	(0.164)	(0.159)	(0.096)	(0.164)	(0.189)
Number of friends who are smokers	0.757***	0.857***	0.448***	0.757***	-0.020
	(0.040)	(0.039)	(0.022)	(0.040)	(0.339)
Number of observations	8,148	8,150	8,148	8,148	
Pseudo R^2	0.213		0.214	0.213	

Note:
1. The z statistics of the coefficients are in parentheses.
2. * $p < .10$, ** $p < .05$, *** $p < .01$

Table A6. Results of regression of choice of low-price versus high-price brands

	Logit	Panel-logit	Probit	Panel-probit	IV probit
Price of low-price brands per pack of	0.001	-0.001	0.000	-0.000	0.018
20 cigarettes (taka in 2015 prices)	(0.003)	(0.004)	(0.001)	(0.002)	(0.136)
Price of high-price brands per pack of	0.001	0.002	0.000	0.001	0.004
20 cigarettes (taka in 2015 prices)	(0.002)	(0.002)	(0.001)	(0.001)	(0.011)
Household income (taka in 2015	-0.028***	-0.035***	-0.016***	-0.020***	-0.021
prices)	(0.003)	(0.003)	(0.001)	(0.002)	(0.022)
Age (years)	0.031***	0.039***	0.016***	0.021***	0.017***
	(0.004)	(0.005)	(0.002)	(0.003)	(0.002)
Education (Reference: illiterate)					0.000
					(.)
1 to 8 years	-0.540***	-0.755***	-0.249**	-0.410***	-0.303
	(0.196)	(0.200)	(0.098)	(0.109)	(0.336)
9 years or more	-1.610***	-2.328***	-0.856***	-1.297***	-0.950**
	(0.213)	(0.219)	(0.109)	(0.120)	(0.401)
Occupation (Reference: owner farmer)					0.000
					(.)
Tenant farmer	-0.908***	-1.063***	-0.447**	-0.584***	-0.436
	(0.345)	(0.365)	(0.187)	(0.202)	(0.368)
Self-employed in non-farm	-0.742***	-0.793***	-0.345***	-0.415***	-0.382**
agricultural activity	(0.205)	(0.225)	(0.109)	(0.122)	(0.166)
Self-employed in non-	0.532	0.275	0.270	0.134	0.190
agricultural activity	(0.371)	(0.379)	(0.173)	(0.197)	(0.303)
Farm wage laborer	0.866*	0.796	0.463**	0.421	0.418
	(0.469)	(0.525)	(0.230)	(0.279)	(0.516)
Non-farm agricultural	-0.355	-0.330	-0.121	-0.157	-0.136
wage laborer	(0.231)	(0.243)	(0.121)	(0.132)	(0.143)
Non-agricultural wage	-1.806***	-2.193***	-0.978***	-1.216***	-1.130
laborer	(0.425)	(0.429)	(0.246)	(0.241)	(0.715)
Professional (e.g.,	-1.573***	-1.862***	-0.857***	-1.031***	-0.935***
physician, engineer)	(0.275)	(0.287)	(0.153)	(0.159)	(0.245)
Managerial, administrative, or	-0.937**	-1.493***	-0.489**	-0.818***	-0.590
clerking	(0.370)	(0.408)	(0.206)	(0.226)	(0.780)
Student	-0.713***	-1.021***	-0.359**	-0.559***	-0.340*
	(0.267)	(0.295)	(0.151)	(0.163)	(0.189)
Unemployed	-0.031	-0.420	0.030	-0.229	-0.016
	(0.410)	(0.556)	(0.235)	(0.303)	(0.343)
Housewife/Housekeeper/	-0.879***	-0.990***	-0.434***	-0.535***	-0.472**
Household manager	(0.201)	(0.213)	(0.106)	(0.115)	(0.220)
Resident of urban area (Reference:	-0.852***	-1.696***	-0.470***	-0.954***	-0.489***
rural area)	(0.117)	(0.145)	(0.066)	(0.082)	(0.119)
Married	0.383***	0.506***	0.226***	0.292***	0.236**
	(0.141)	(0.149)	(0.078)	(0.084)	(0.097)
Number of friends who are smokers	-0.017	0.019	-0.006	0.012	0.011
	(0.037)	(0.042)	(0.021)	(0.023)	(0.091)
Number of observations	5,961	5,962	5,942	5,962	5,942
Pseudo R^2	0.256		0.254		

Notes:
1. The z statistics of the coefficients are in parentheses.
2. * $p < .10$, ** $p < .05$, *** $p < .01$
3. The Wald statistics from IV probit regression using composite housing index as an instrument for the cigarette price for all brands are not statistically significant. Hence, the exogeneity of price is not rejected.

Table A7. Results of regressions of the number cigarettes smoked per day

	OLS for low-price brands	OLS for high-price brands	2SLS for low-price brands	2SLS for low-price brands
Price of low-price brands per pack of 20 cigarettes (taka in 2015 prices)	-0.014<br>(0.016)	0.018<br>(0.065)	0.320<br>(0.292)	-0.125<br>(1.229)
Price of high-price brands per pack of 20 cigarettes (taka in 2015 prices)	0.041***<br>(0.010)	-0.011*<br>(0.006)	0.088<br>(0.076)	-0.164<br>(0.290)
Household income (taka in 2015 prices)	-0.006<br>(0.014)	0.025<br>(0.018)	-0.090<br>(0.086)	0.132<br>(0.117)
Age (years)	0.024<br>(0.019)	0.006<br>(0.029)	0.042<br>(0.027)	0.026<br>(0.103)
Education (Reference: illiterate)				
1 to 8 years	-0.961*<br>(0.553)	-1.024<br>(1.253)	-1.105<br>(0.871)	0.806<br>(2.463)
9 years or more	-1.573**<br>(0.637)	-1.563<br>(1.198)	-3.344*<br>(1.813)	1.367<br>(4.736)
Occupation (Reference: owner farmer)				
Tenant farmer	0.032<br>(1.132)	0.930<br>(1.426)	-2.329<br>(1.722)	1.674<br>(3.085)
Self-employed in non-farm agricultural activity	0.123<br>(0.686)	1.335<br>(1.231)	-1.351<br>(1.482)	1.820<br>(3.349)
Self-employed in non-agricultural activity	-0.576<br>(0.922)	-1.036<br>(1.392)	-1.888<br>(2.262)	2.208<br>(9.276)
Farm wage laborer	0.463<br>(1.072)	2.443<br>(1.695)	-0.827<br>(1.228)	-0.056<br>(3.309)
Non-farm agricultural wage laborer	0.424<br>(0.805)	2.537<br>(2.085)	-0.210<br>(0.846)	1.578<br>(3.606)
Non-agricultural wage laborer	0.459<br>(1.329)	0.041<br>(1.360)	-5.082<br>(3.689)	1.782<br>(8.016)
Professional (e.g., physician, engineer)	-0.741<br>(0.945)	2.063<br>(1.350)	-1.801<br>(2.631)	3.741<br>(6.002)
Managerial, administrative, or clerking	-0.969<br>(1.188)	-0.472<br>(1.410)	-2.732<br>(3.085)	-1.353<br>(11.838)
Student	0.019<br>(0.872)	0.976<br>(1.283)	0.337<br>(1.145)	-2.299<br>(6.420)
Unemployed	-2.713***<br>(1.007)	1.415<br>(2.278)	-4.310***<br>(1.600)	-0.253<br>(3.061)
Housewife/Housekeeper/Household manager	1.137<br>(0.719)	1.908*<br>(1.060)	-0.680<br>(1.362)	1.633<br>(6.629)
Resident of urban area (Reference: rural area)	-1.689***<br>(0.385)	-1.176<br>(0.916)	-1.762**<br>(0.811)	0.372<br>(4.594)
Married	-0.025<br>(0.771)	0.900<br>(0.968)	0.711<br>(0.676)	0.997<br>(3.070)
Number of friends who are smokers	0.944***<br>(0.179)	0.217<br>(0.330)	1.231***<br>(0.345)	-0.091<br>(0.558)
Number of observations	4,404	1,427	4,405	1,427
